# Commentary: The Flavonoid Baicalein Rescues Synaptic Plasticity and Memory Deficits in a Mouse Model of Alzheimer’s Disease

**DOI:** 10.3389/fneur.2016.00141

**Published:** 2016-08-29

**Authors:** Salvatore Chirumbolo, Geir Bjørklund

**Affiliations:** ^1^Department of Neurological and Movement Science, University of Verona, Verona, Italy; ^2^Council for Nutritional and Environmental Medicine, Mo i Rana, Norway

**Keywords:** Alzheimer disease, synaptic loss, GSK-3β, baicalein, flavonoids, astrocyte, glutamate receptors

A recent article by Gu et al. reported that the long-term oral administration of baicalein in mouse, inhibited the activity of lipooxygenase 12/15 LXO and glycogen synthase kinase 3β (GSK-3β) in hippocampal slices, also reducing the activity of β-secretase enzyme (BACE1) and the concentration of total beta-amyloid proteins (Aβ), leading to the *in vivo* restoration of spine number, synaptic plasticity, and memory deficits (1).

Baicalein (5,6,7-trihydroxy-2-phenyl-chromen-4-one) is present in the plant genus *Scutellaria*, e.g., *Scutellaria baicalensis*, the flavone recently went in the spotlight for its ability in preventing Alzheimer disease (AD) and other neurodegenerative disorders (2–4). According to the authors, baicalein is able to prevent the impairment in the hippocampal long-term potentiation (LTP) induced by β-amyloids (Aβ) and improve cognitive deficit associated with AD (1). Recently, also metformin exhibited positive effects on AD quite comparable to baicalein, i.e., the inhibition of the activity of Aβ on LTP in a high fat diet (HFD) rat model (5). As evidence about the anti-obesity and anti-diabetes activity of baicalein was reported elsewhere, the activity of metformin may give insights on the role exerted by baicalein in AD. Despite the evidence reported by Asadbegi et al. some controversial result yet exists, as in type 2 diabetes models metformin increases the expression of the β-amyloid precursor protein (APP), through the activation of NF-κB, causing accumulation of Aβ in rat brain (5, 6). Whether metformin has either a beneficial or a noxious effect depending on the cellular system considered or if it acts through a typical bimodal mechanism, a hallmark shared by several bioflavonoids, is yet far to be fully elucidated. As like as flavonoids, even metformin, which is a well known anti-diabetic drug, possesses anti-oxidant and anti-inflammatory properties (7). This recent evidence suggested us a possible mechanism for baicalein action in AD prevention, as also for the flavonoid baicalein, a bimodal activity might be addressed, at least as concern its action on oxidative stress and metabolism. The main advantage respect to metformin is that baicalein is a plant-derived natural compound. As many bioflavonoids, baicalein inhibits phosphorylation of many fundamental signaling protein kinases, leading to its typical anti-inflammatory activity, e.g., it inhibits the ERK/MAPK signaling cascade, acting on the phosphorylation of MEK-1 by Raf-1 and inducing dampening of NF-κB (8). This anti-phoshorylation property was reported also by Gu et al. as baicalein should exert its action by preventing the phosphorylation of tau protein in APP/PS1 mice (1). Still, a metabolic, anti-oxidant activity from baicalein, besides its targeting glutamatergic neurotransmission, may explain some evidence reported (1).

The authors showed that Aβ binding to synapse-related sites mediates the reduction of synaptic loss and also modulates the damage of the glutamatergic synaptic transmission, while baicalein can prevent this damage (1). Furthermore, the paper by Gu et al. would suggest whether the mechanism by which baicalein acts on AD may resemble some other neuro-protective compound, in order to focus onto commonly shared targets. Curiously, as like as metformin, also baicalein succeeded in rescuing hippocampal LTP impaired by the action of Aβ and the authors suggested a mechanism working through the PI3K signaling pathway (1, 5). Further bioflavonoids seem to have a comparable effect on the synaptic failure induced by Aβ, e.g., the glycosylated-flavonol troxerutin, is able also to reduce impairments in cognitive function caused by metabolic syndrome or HFD (9, 10) and the flavone nobiletin, from *Citrus* fruit, rescued cognitive function and memory impairment in animal fear conditioning tests by reverting the inhibition caused by Aβ on the membrane trafficking of glutamate receptors, which is required for LTP (11). And even nobiletin has an impact on obesity and metabolic syndrome (12). The question is if there is a fundamental connection between metabolic syndrome and neurodegenerative disorders in the reported action of baicalein.

A possible mechanism linking these effects and shared by metformin besides flavonoids might involve their indirect action on the impaired insulin signaling and glucose metabolism, which causes AD in brain (13). For example, the paper by Gu et al. showed that baicalein inhibits the activity of GSK-3β. Brain derived insulin is expressed by the activity of the transcription factor neurogenic differentiation 1 (NeuroD1) and inhibitors of GSK-3β increase insulin gene (Ins2) and NeuroD1 in the mouse brain, through a Wnt/β-catenin pathway (14). Gu et al. also reported that baicalein increased the *N*-methyl-d-aspartate glutamate receptor (NMDAR)-mediated LTP in rat hippocampus and it is well known that glutamate, which is spontaneously released by astrocytes, is involved in the setting of a LTP threshold (15). Therefore, it is possible that an activity of baicalein described by Gu et al. may target the insulin signaling by inhibiting the GSK-3β pathway.

The most acknowledged activity of plants bioflavonoids is their targeting oxidative stress, gene transcription factors involved in the cellular response to oxidative stress and mechanisms involved in the scavenging of oxidative stressors. Actually, it is not surprising that for the interesting evidence reported by Gu et al. the anti-oxidant property of baicalein might even play a major role. For example, the activity of membrane PI3K in astrocytes, through the up-regulation of the signaling mechanism, involving the activation of phospholipase C and protein kinase C-delta (PKC-δ), causes a weak but persistent stimulation of the N-methyl-d-aspartate receptors (NMDARs) from astrocytes and this mechanism is coupled with the phosphorylation at Thr(395), Ser(433), and Thr(439) by PKC-δ of the nuclear factor-(erythroid-derived 2)-like 2 (Nrf2), then linking glutamate transmission with anti-oxidant activity and neuron survival. Translocation to the nucleus of Nrf2 enhances the NMDAR stimulation (16). Baicalein may target this mechanism, in the system described by Gu et al. This should suggest that a possible effect of baicalein may be exerted also on the glia, besides neurons, particularly during the inflammatory response (17). In this circumstance, further research should address the widespread opinion that plant polyphenols act on more than one level, fundamentally by targeting signaling system that are involved in cell survival and stress response. This would involve the existence of complex machinery encompassing both homeostasis of the energetic balance and cell response to stressors. In this sense, the bio-molecular mechanism by which baicalein caused the evidence reported by Gu et al. may mainly depend on its anti-oxidant property, as β-secretase 1 (BACE1) activity, which increases the level of APP, is promoted by pro-oxidant factors (18). This potential should shed a light also on the neuroprotective role of those natural compounds, which are recognized as anti-diabetic and obesity-preventive molecules.

## Author Contributions

SC conceived the paper, SC and GB read the literature, GB contribute in the editing of the document, SC wrote with GB the manuscript, SC and GB revised the first draft, SC submitted the manuscript.

## Conflict of Interest Statement

The authors declare that the research was conducted in the absence of any commercial or financial relationships that could be construed as a potential conflict of interest.
